# Obituaries

**Published:** 1879-03-20

**Authors:** 


### *OBITUARIES.*

Dr. T. CHALMERS DOW, Professor of Gynæcology in the Nashville Medical College, died on Tuesday, January 7th, 1879.

Dr. ROBERT C. FOSTER, of Nashville, died on Januaay 14th, 1879.

One of our subscribers, a true and good man, Dr. GEORGE A. DYER, aged 50 years, died of pneumonia at Washington, Davies County, Ind., on the 12th of January, 1879.

---

### *CINCHONIA ALKALOID.*

The Cinchonia Alkaloid introduced to the Profession by Messrs. Powers & Weightman, of Philadelphia, and advertised in our last issue, is meeting with great favor, and is likely to come into very general use.

---

### *CASCARA SAGRADA.*

Be sure to examine this article as advertised in this number by the enterprising House of Parke, Davis & Co.

